# Screen Printed Based Impedimetric Immunosensor for Rapid Detection of *Escherichia coli* in Drinking Water

**DOI:** 10.3390/s20010274

**Published:** 2020-01-03

**Authors:** Martina Cimafonte, Andrea Fulgione, Rosa Gaglione, Marina Papaianni, Rosanna Capparelli, Angela Arciello, Sergio Bolletti Censi, Giorgia Borriello, Raffaele Velotta, Bartolomeo Della Ventura

**Affiliations:** 1 Department of Physics “Ettore Pancini”, University of Naples “Federico II”, Via Cinthia, 26, 80126 Naples, Italy; martina.cimafonte@unina.it (M.C.); rvelotta@unina.it (R.V.); 2 Istituto Zooprofilattico Sperimentale del Mezzogiorno, Via Salute, 2, 80055 Portici Naples, Italy; andrea.fulgione@unina.it (A.F.); giorgia.borriello@cert.izsmportici.it (G.B.); 3 Department of Agriculture, University of Naples “Federico II”, Via Università, 133, 80055 Portici Naples, Italy; marina.papaianni@unina.it (M.P.); capparel@unina.it (R.C.); 4 Department of Chemical Sciences, University of Naples “Federico II”, Via Cinthia, 26, 80126 Naples, Italy; rosa.gaglione@unina.it (R.G.); angela.arciello@unina.it (A.A.); 5 Cosvitech Società Consortile a Responsabilità Limitata, 80142 Naples, Italy; sergiobolletti@cosvitec.eu; 6 Department of Physics, Politecnico di Milano, Piazza Leonardo da Vinci, 32, 20133 Milano, Italy

**Keywords:** *Escherichia coli*, immunosensor, electrochemical impedance spectroscopy, antibodies, photochemical immobilization technique, cyclic voltammetry

## Abstract

The development of a simple and low cost electrochemical impedance immunosensor based on screen printed gold electrode for rapid detection of *Escherichia coli* in water is reported. The immunosensor is fabricated by immobilizing anti-*E. coli* antibodies onto a gold surface in a covalent way by the photochemical immobilization technique, a simple procedure able to bind antibodies upright onto gold surfaces. Impedance spectra are recorded in 0.01 M phosphate buffer solution (PBS) containing 10 mM Fe(CN)_6_^3−^/Fe(CN)_6_^4−^ as redox probe. The Nyquist plots can be modelled with a modified Randles circuit, identifying the charge transfer resistance *R_ct_* as the relevant parameter after the immobilization of antibodies, the blocking with BSA and the binding of *E. coli*. The introduction of a standard amplification procedure leads to a significant enhancement of the impedance increase, which allows one to measure *E. coli* in drinking water with a limit of detection of 3 × 10^1^ CFU mL^−1^ while preserving the rapidity of the method that requires only 1 h to provide a “yes/no” response. Additionally, by applying the Langmuir adsorption model, we are able to describe the change of *R_ct_* in terms of the “effective” electrode, which is modified by the detection of the analyte whose microscopic conducting properties can be quantified.

## 1. Introduction

Water is a natural resource, essential for the life sustainment and significant for health in both developing and developed countries worldwide. In the recent years, the inappropriate handling of urban, industrial and agricultural wastewater has affected the quality of the drinking water which turns out to be alarmingly contaminated and chemically polluted [1]. Contaminated water causes a serious impact on the population as it induces a large number of diseases caused by microorganisms [2]. According to the World Health Organization (WHO), water-related diseases are a worldwide problem and each year 3.4 million people, mostly children below the age five, suffer from waterborne diseases and die [3]. Pathogenic bacteria in water are mainly responsible for human infection diseases and one of the most common bacteria associated with the sanitary risk of water is the species *Escherichia coli*.

*Escherichia coli* is a gram-negative bacterium of the genus *Escherichia* identified for the first time in 1885 by the German paediatrician and bacteriologist Theodor Escherich. *E. coli* is a rod-shaped gut bacterium, natural inhabitant in the intestinal tracts of humans and warm-blooded animals. It is considered one of the most dangerous pathogens because some strains can cause serious illness, including severe diarrhoea, urinary tracts infections, inflammations and peritonitis. As a consequence, the presence of *E. coli* in drinking water is considered as a possible indicator of the microbiological water quality deterioration and the presence of *E. coli* in processed food products can indicate faecal contamination [1]. In fact, according to WHO and the European Union [4] no *E-coli* should be detected in 100 mL of water. Such a limit can only be reached by time-consuming measurements carried out in equipped laboratories; therefore, nowadays one of the challenges in food industry and environmental monitoring is the development of methods for the rapid detection of low levels of *E. coli*

Conventional methods for the detection of *E. coli* include multiple-tube fermentation, membrane filter and plate counting. Although, these culture-based methods are accurate, reliable and have low detection limits, they are typically labor-intensive and time-consuming since they require 2–3 days to yield initial results and up to 7–10 for the confirmation [5]. Other detection methods, such as ELISA [6] and PCR [7,8] are less time consuming but they require expensive equipment and initial sample pre-treatment which make the application of these methods limited only to the laboratory environment [9,10,11]. Thus, the research for new strategies that could be promising alternatives to the conventional techniques to be used in industrial applications is very timely.

Detection techniques based on biosensors are widely recognized as powerful tools for the detection of bacteria due to their several advantages such as fast response, robustness, low cost, sensitivity, specificity and real time detection [12]. Among them, biosensors based on antibody-antigen interaction (the so-called immunosensors) are broadly investigated, and, in fact, immunosensors using electrochemical [13], surface plasmon resonance (SPR) [14], piezoelectric [15] and cantilever [16] based transducers have been applied for *E. coli* detection. Electrochemical biosensors are considered powerful instruments overcoming the limitations of the conventional methods due to their multiple advantages such as low cost, high sensitivity, fast response, robustness and simple operation [17,18,19]. Among different electrochemical techniques, electrochemical impedance spectroscopy (EIS) is very commonly used to investigate the recognition events at electrode/electrolyte interface [11,20] and EIS based biosensors are particularly attractive since they allow antigen detection with high sensitivity. In the last decade, different impedimetric immunosensors for the detection of *E. coli* have already been developed [21,22,23,24].

The immobilization of antibodies (Abs) is a crucial step in the realization of an immunosensor because its analytical performance strongly depends both on the orientation of the antibodies and their density on the surface. Thus, it would highly desirable to rely on a surface functionalization procedure that would overcome such an issue [25,26]. Generally, antibodies can be immobilized via physical or chemical adsorption involving electrostatic or ionic bonds, hydrophobic interactions and van der Waals forces [27,28], via covalent attachment [29,30,31,32], by using the biotin–avidin approach [33,34] or immobilizing intermediate binding proteins, such as protein A or G [35,36,37,38] and through entrapment into a polymer matrix [39,40,41,42]. These approaches, particularly protein A and G method, are time-consuming, but even more important, require a surface modification or pre-treatment for an effective protein A/G binding [43] that can affect the robustness and reproducibility of the protocol.

Among all the possible strategies, self-assembled monolayers (SAMs) is currently one of the most widespread methods for electrode functionalization aiming at detecting *E. coli* by electrochemical approaches. For instance, an oriented anti-*E. coli* immobilization on gold electrode surfaces could be achieved by exploiting SAMs of thiolated carboxylic acid [44,45,46] or by immobilizing anti-*E. coli* on electrochemically deposited cysteamine layers [45]. The use of thiolated scaffolds such as protein G [45,47] and cross-linkers as glutaraldehyde [48], the latter allowing the immobilization of anti-*E. coli* onto electrochemically synthesized polyaniline substrate, have also been reported with promising results for the immobilization of anti-*E. coli*.

Thus, SAMs are broadly used as linkers for the immobilization of antibodies onto gold electrode surfaces, but in spite of the many advantages they offer in many applications, there are several issues that should be considered in order to find out and control their physical and chemistry properties [49,50,51]. SAMs on gold surfaces are usually represented as perfect monolayers, with molecules in a closed packed configuration. Nevertheless, this concept is far from reality and the control of the quality of SAMs is a key point in many applications. The realization of a well-assembled monolayer strongly relies on the purity of the solutions used and the presence of even a low amount of contaminants, as for instance thiolated precursor molecules that are the typical impurities in thiol compounds, can lead to a non-uniform and, hence, non-ideal monolayer [52]. In addition, the electrode surface plays an important role in the realization of SAMs. EIS analysis of electrode surfaces with different roughness showed that the rougher substrate exhibited small and variable response as a result of a non-ideal SAM formation, while the smoother surface produces higher and more reproducible response due to the increase of the SAM homogeneity [53]. Moreover, over the years several studies have worked to clarify the true nature of the interaction gold-thiols SAMs [36,54,55,56]. Considering these issues, it is worth developing alternative methods to covalent bind antibodies on gold electrode surfaces in an easier, more rapid and reliable way.

In this paper, we propose a simple and low-cost EIS immunosensor based on screen printed gold electrodes (AuSPEs) for the detection of *Escherichia coli*. The anti-*E. coli* antibodies were immobilized on the gold electrode surface using the Photochemical Immobilization Technique (PIT) a simple procedure able to steer antibodies in a convenient orientation of the Fab region once immobilized onto gold surface [57,58]. In this work, PIT has been used for the first time in the functionalization of commercial gold electrodes in order to develop an “on-off” electrochemical immunosensor based on impedance spectroscopy (EIS) using Fe(CN)_6_^3−^/Fe(CN)_6_^4−^ as redox probe. The effectiveness of our approach is demonstrated by detecting *E. coli* ATTCC 25922 in drinking water. Our immunosensor exhibits a limit of detection (LOD) of 3 × 10^1^ CFU mL^−1^, with no need for pre-concentration and pre-enrichment steps. The selectivity against other bacteria was evaluated and the immunosensor was applied to the analysis of inoculated drinking water samples.

## 2. Materials and Methods

### 2.1. Chemicals and Materials

Gold screen printed electrodes (AuSPEs) were purchased from BVT Technologies (Strážek, Czech Republic). They include a gold disk-shaped (*d* = 1 mm) working electrode, a silver/silver chloride electrode and a gold counter electrode, all of them printed on a corundum ceramic base (0.7 cm × 2.5 cm). All potential values were referred to the silver/silver chloride reference electrode. Phosphate buffer solution (PBS) was prepared by dissolving PBS tablets (from GoldBio, St Louis, MO, USA) in Milli-Q water (each tablet prepares 100 mL of a 0.01 M PBS solution). Anti-*E. coli* polyclonal antibody (5.5 mg mL^−1^) was obtained from Thermo Fisher Scientific (Rockford, IL, USA) and anti-*E. coli* solutions (25 μg mL^−1^) were prepared in a 0.01 M PBS solution (pH 7.4). Bovine serum albumin (BSA), potassium hexacyanoferrate (II) trihydrate (K_4_Fe(CN)_6_·3H_2_O), potassium hexacyanoferrate (III) (K_3_Fe(CN)_6_) and sulphuric acid (H_2_SO_4_ 98%) were purchased from Sigma-Aldrich (Milano, Italy). The microfluidic setup involves a fluidic cell, silicon tubes and a continuous pump (HNP Mikrosysteme GmbH, Schwerin, Germany). The total volume of the circuit is about 100 μL, the cell volume is about 10 μL and the flow rate is 6 μL s^−1^.

### 2.2. Apparatus

Electrochemical impedance spectroscopy (EIS) and cyclic voltammetry (CV) measurements were carried out with a potentiostat/galvanostat and impedance analyzer PALMSENS (Utrecht, The Netherlands) model PalmSens3 controlled by a computer through the PSTRACE version 5 software. Cyclic voltammetry (CV) and electrochemical impedance spectroscopy (EIS) were conducted in the presence of Fe(CN)_6_^3−^/Fe(CN)_6_^4−^ (1:1, 10 mM) as redox probe in 0.01 M PBS solution (pH = 7.4). In CVs, potential was cycled from −0.6 V to 0.6 V with a scan rate of 0.15 V s^−1^ in 10 mM Fe(CN)_6_^3−^/Fe(CN)_6_^4−^. EIS measurements were performed at the frequency range from 5 Hz to 10000 Hz at the formal potential of 0.16 V and using an amplitude perturbation of 10 mV. The impedance data were shown in the Nyquist plot and the EIS spectrum Analyzer software, supplied with the instrument, was used to fit EIS data to the electrical equivalent circuit in order to obtain the fit-component parameters values.

**Figure 1:**
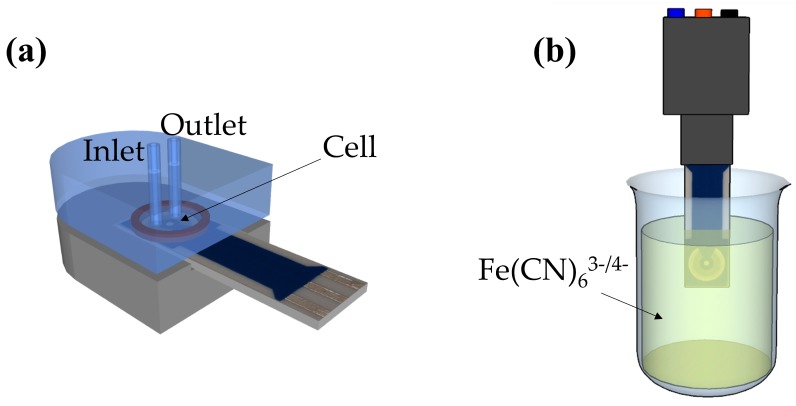
(**a**) Sketch of the fluidic cell used for an effective interaction of the solutions (Abs, BSA and *E. coli*) with the electrode and (**b**) scheme used for the electrochemical measurements: the AuSPE was dipped in a beaker containing 1.5 mL of Fe(CN)_6_^3−/4−^ and it was connected to the potentiostat through a holder (in grey) for the impedance/current measurements.

**Scheme 1:**
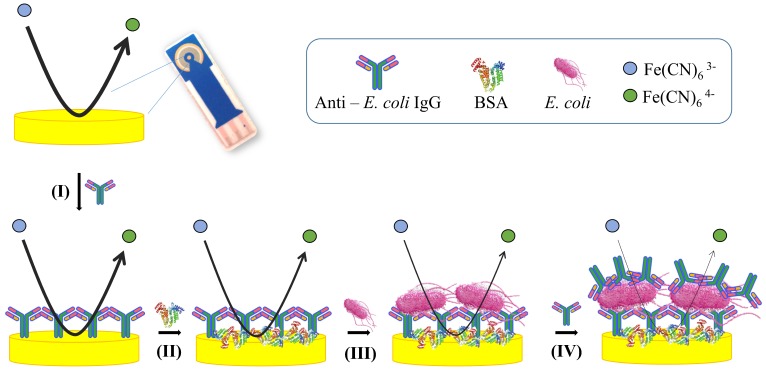
Schematic diagram (not in scale) of the stepwise functionalization and detection. The black line represents the intensity of the redox reaction, which is inhibited as the surface covering grows. The reduction of its thickness is associated to a decrease of the “effective” area available for the electrolyte current, which is measured as an increase of the charge transfer resistance.

A fluidic setup including a Plexiglas cell, silicon tubes and a continuous pump was used for the flowing of the different solutions (Abs, BSA and *E. coli*). A schematic representation of the cell is shown in Figure 1a. The main feature of the cell is its small volume that facilitate the interaction of the particles in the solutions (Abs, BSA, *E. coli*, etc.) with the electrode. Any solution was conveyed by a continuous pump at a flow rate of 6 µL s^−1^. Although effective for the interaction, such a cell was unsuitable for electrochemical measurements in view of the small amount of electrolytes involved. Thus, after each interaction (i.e., each steps shown in Scheme 1), we took the electrode out of the cell and dipped it into a 1.5 mL beaker containing Fe(CN)_6_^3−^/Fe(CN)_6_^4−^ (Figure 1b), in which the electrochemical measurements were carried out at room temperature.

### 2.3. Preparation of the Biological Sample

Bacterial strain *E. coli* ATCC 25922 was grown in Muller Hinton Broth (MHB, Becton Dickinson Difco, Franklin Lakes, NJ, USA) and on Tryptic Soy Agar (TSA; Oxoid Ltd., Hampshire, UK). In all the experiments, bacteria were inoculated and grown overnight in MHB at 37 °C. The next day, bacteria were centrifuged and solubilized in drinking water at the desired cell densities (10^1^–10^6^ CFU mL^−1^). By colony counting assays, it was verified that bacterial growth was negligible in PBS 1X with respect to MHB through a time interval of 3 hrs at room temperature, whereas bacterial death was not observed. Clinical isolated bacteria *Salmonella enteriditis 706 RIVM* [59] and *Acinetobacter baumannii* (ATCC 17978) were grown in the same way and diluted drinking water in order to verify the specificity of the immunosensor towards *E. coli* in comparison with non-target bacteria.

### 2.4. UV Activation of Antibody Solution

**Figure 2:**
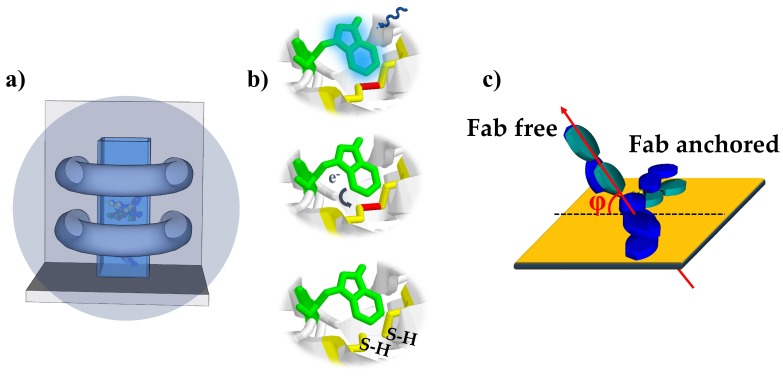
(**a**) UV lamp used for the UV activation of the antibodies. Emitting wavelength: 254 nm, diameter of each lamp: 1.5 cm, dimensions internal: space 1 cm × 1 cm × 4 cm, power of each lamp 6 W, irradiation intensity 0.3 W/cm^2^; (**b**) Description of the reaction involved in PIT: the UV photon energy is adsorbed by tryptophan and transferred to near cys-cys. The result is the cleavage of the disulphide bridges and the formation of new thiol (SH) groups; (**c**) Antibody is immobilized onto the surface so that the angle φ is 45° on average thereby providing one Fab free and well-exposed to the environment.

The gold SPE was functionalized with anti-*E. coli* antibodies, previously activated by the Photochemical Immobilization Technique (PIT) [57,58]. PIT is a functionalization method able to tether antibodies (Abs) upright on metal (gold or silver) surfaces with their binding sites well exposed to the environment [60], based upon the selective photochemical reduction of disulphide bridges in immunoglobulins (IgGs) produced by UV activation of near aromatic amino acid [61]. Briefly, a selective photoreduction of disulphide bridges produced by the UV activation of the trp/cys-cys triad occurs. This triad is a typical structural feature of IgGs and basically, the UV photon energy is adsorbed by tryptophan and transferred to near electrophilic species like the close cys-cys. The result is the cleavage of the disulphide bridges and the formation of new thiol (SH) groups able to bind, through a covalent bond, thiol reactive surfaces like gold ones (Figure 2b). Every IgGs have twelve triads but, it has recently been demonstrated [57] that only two of them are involved in this process. These triads are located in the constant variable region and allow the attack of the antibody to the surface with one of the two Fab regions exposed to the solution (Figure 2c) [57]. Considering that the triad of residues trp/cys-cys can be found in every IgGs, the PIT is applicable in a wide range of fields. PIT is accomplished by activating 300 μL of antibody samples (25 μg mL^−1^) in a quartz cuvette by two low pressure mercury lamp (LP Hg lamp) emitting at 254 nm and manufactured by Procom Alta Tecnologia s.r.l. (Dicomano, FI, Italy) (Figure 2a). The two lamps (1.5 cm of diameter) are horseshoe-shaped and mounted in a stacked configuration so that its internal empty space fits with a (quartz) cuvette whose dimensions are 1 cm × 1 cm × 4 cm. The power of each lamp is 6 W and by considering that the cuvette containing the anti-*E. coli* IgG solution is close to the lamps, the effective irradiation intensity used for the antibody activation is about 0.3 W/cm^2^. The samples are irradiated for 30 s. This time is the result of an optimized protocol that, as confirmed by the Ellman’s assay [62], produces an high concentration of activated Abs while guaranteeing no denaturation of antibodies as evidenced by their efficiency in antigen binding in the developed biosensors.

After the activation, the Abs solution was conveyed to the electrode surface by means of a fluidic circuit. In previous works, this method has been used in a number of experiments to develop sensitive and selective QCM-based immunosensors [63,64,65,66,67] as well as colorimetric biosensors [68].

### 2.5. Immunosensor Development and E. coli Detection

Before the functionalization, the AuSPE was electrochemically cleaned by applying 10 cycles between −0.4 V and 1.4 V at a scan rate of 0.1 V s^−1^ in 0.1 M H_2_SO_4_. The electrode was rinsed with a copious amount of Milli-Q water and it was ready to use. The cleaned SPE was placed in the fluidic cell and the experimental procedure for impedance measurements consists in the flowing of different solutions onto the sensitive gold surface of the AuSPE. First, a solution of UV-activated anti-*E. coli* antibodies (25 μg mL^−1^) was conveyed onto the gold sensitive surface for 15 min by applying a constant flow rate (6 μL s^−1^). Since the Ab activation only lasts approximately five minutes [57], to saturate the gold electrode surface, such a step was repeated 4 times with a fresh irradiated Abs solution. Subsequently, the electrode was rinsed for 1 h with 0.01 M PBS buffer to remove the unbound antibodies. After that, a BSA solution (50 μg mL^−1^) flowed into the cell for 15 min filling the remaining free space on the gold surface. This blocking step is crucial because, by filling the free remaining spaces on the gold electrode surface, the possible non-specific interactions of the following molecules are avoided. A 1mL aliquot of drinking water incubated with different concentration of *E. coli* cells (from 10^1^ to 10^8^ CFU mL^−1^) flowed into the circuit for 30 min at room temperature and the bacteria cells were captured by the immobilized antibodies. The electrode was rinsed with 0.01 M phosphate buffer to remove non-specifically and weakly bound bacteria for 5 min. Each detection was repeated three times. The difference in impedance measured before and after *E. coli* incubation, normalized with the impedance value obtained after Abs immobilization, was taken as the signal produced by the binding between immobilized antibodies and target bacterial cells. An aliquot of 1 mL of drinking water, without incubation of bacteria, was used as negative control.

### 2.6. Enhanced Sensitivity Protocol

In order to improve the signal, an amplification step has been included in the experimental procedure. The response enhancement has been achieved by conveying anti-*E. coli* (25 μg mL^−1^) into the microfluidic cell for 30 min. This additional step, which leads to the formation of a sandwich complex, was used to amplify the slight impedance increment obtained after *E. coli* detection at low concentration. In such a case, the formation of a sandwich complex further hinders the electron-transfer process thus improving the electrochemical response. A final washing phase with 0.01 M PBS is used to remove unbound or weakly bonded molecules.

## 3. Results and Discussion

### 3.1. Principle of the Impedimetric Biosensor

The different steps involved in the preparation of our immunosensor are schematically illustrated in Scheme 1 (not to scale). After cleaning the AuSPE with H_2_SO_4_, the surface is functionalized (step I) with previously activated antibodies using the LP Hg lamp above described. Then, bovine serum albumin (BSA) is used as blocking reagent to fill the free remaining spaces on the gold electrode (step II). In the next step (step III) a specific binding event occurs between the immobilized antibodies and the *E. coli* cells. Finally, in order to improve the sensitivity, a sandwich complex is realized by conveying a fresh anti-*E. coli* Abs solution to the circuit. Consecutive steps of the immunosensor development as well as the *E. coli* detection are described in detail in the Materials and Methods section above.

**Figure 3:**
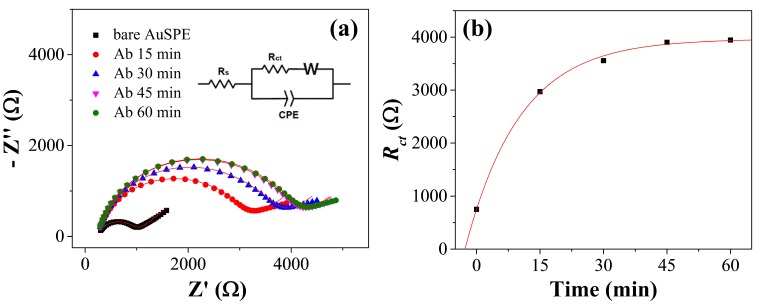
(**a**) EIS spectrum measured at different times while the UV irradiated antibodies are conveyed to the interaction cell; (**b**) *R_ct_* as a function of time showing that the surface saturation takes place within one-hour time. The errors of *R_ct_* are within the thickness of the experimental points.

**Figure 4:**
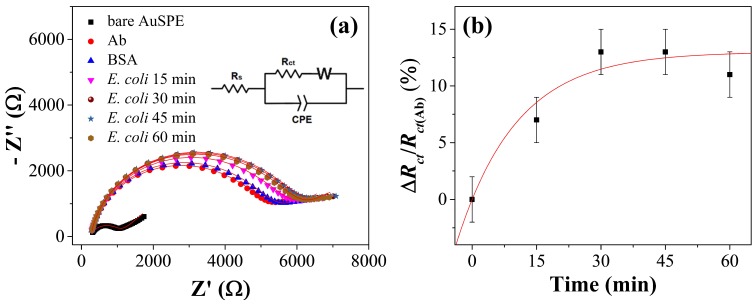
(**a**) EIS spectrum measured at different times with *E. coli* at 10^5^ CFU mL^−1^; (**b**) *R_ct_* as a function of time showing an exponential kinetic with a constant time of 14 ± 7 min.

**Figure 5:**
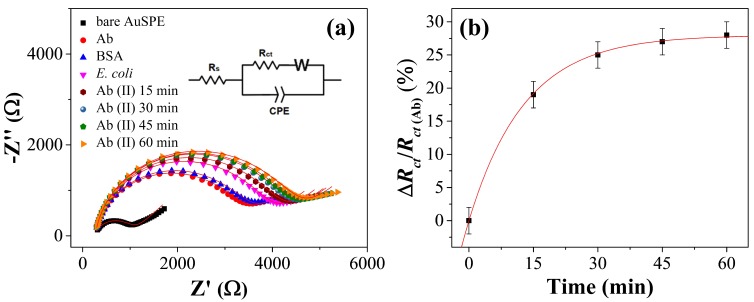
Kinetics of the secondary antibody. (**a**) EIS spectrum measured at different times while a 25 µg mL^−1^ Ab solution is conveyed to the cell after the detection *E. coli* (10^5^ CFU mL^−1^); (**b**) *R_ct_* as a function of time showing an exponential dynamic with a constant time of 13.5 ± 0.3 min.

**Figure 6:**
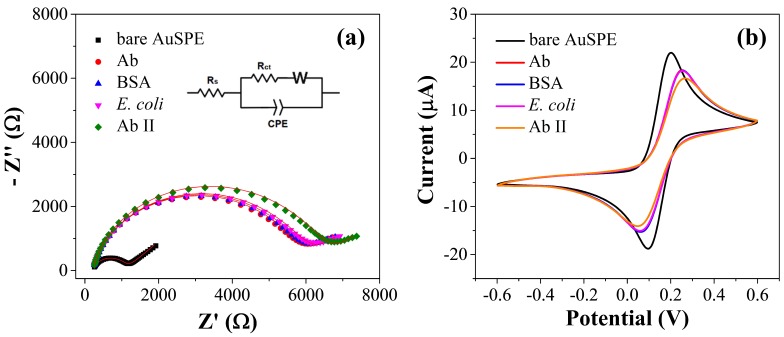
(**a**) EIS and (**b**) CV of the step-by-step immunosensor development and *E. coli* detection in 10 mM Fe(CN)_6_^3−^/Fe(CN)_6_^4−^ solution at pH 7.4.

EIS is characterization techniques to study the electron transfer process and, specifically, the changes of charge transfer resistance caused by the adsorption of isolating molecules to the gold electrode. The impedance data are represented as Nyquist plots (see Figure 3, Figure 4, Figure 5 and Figure 6), where the real and the imaginary components of impedance are plotted in the X and Y axes, respectively. The Nyquist plots consist of two portions: the semicircle portion at high frequencies indicates the electron-transfer process while the linear part at lower frequencies represents the diffusion-limited mass transfer process of the redox probe. A modified Randles circuit (shown as inset in Figure 3, Figure 4, Figure 5 and Figure 6) was used to fit the experimental data over the whole frequency range. In this equivalent circuit, *R_s_* stands for the resistance of the solution, CPE is the constant phase element, *R_ct_* is the charge transfer resistance and W the Warburg impedance. *R_s_* and *W* represent bulk properties of the electrolyte solution and diffusion features of the redox probe in the solution. These parameters are not influenced by modification of the electrode surface and are not modified by the antibodies-bacteria interaction. On the contrary. *R_ct_* depends critically on the dielectric and insulating features at the electrode-electrolyte interface and can be used as sensing parameter since it is very sensitive to electrode modifications. The CPE is introduced in the equivalent circuit instead of a simple capacitor to account for inhomogeneity and defect areas of the layer [69].

### 3.2. Kinetics of the Functionalization and Detection

In the photochemical immobilization technique, the Abs are conveyed to the electrode after they have been irradiated by the UV lamp. Since the activation of the Abs only lasts approximately 5 min [57], it is necessary to optimize the time the solution is fluxed in the cell containing the working electrode (10 µL volume). The results obtained with a flow rate of 6 μL s^−1^ are reported in Figure 3a (see Table S1 for the data), that shows EIS spectra measured at intervals of 15 min, which are required to cover the whole surface. The resulting *R_ct_* is shown in Figure 3b and its behavior with the time is well fitted by an exponential function with time constant of 13 ± 1 min. Thus, the whole functionalization procedure can be considered accomplished in one-hour time. Since no significant change of *R_ct_* is observed after the blocking step, we can assess that the saturation of *R_ct_* shown in Figure 3b corresponds to an electrode fully covered by antibodies.

The kinetic of *E. coli* detection was studied to optimize the performance of the immunosensor. Drinking water samples incubated with a concentration on *E. coli* 10^5^ CFU mL^−1^ was flowed over the antibody-modified electrode and the *R*_ct_ change was monitored at time intervals of 15 min. The results are reported in Figure 4a (Nyquist plot) and 4b (normalized *R_ct_*) with an exponential fit of the experimental data that provides a time constant of 14 ± 7 min (see Table S2 for the data). Although with larger error, such a value is similar to that measured for surface functionalization by Abs suggesting that 30 min was a suitable incubation time to allow the completion of the analyte detection.

We also measured the kinetics of the Ab binding to the *E. coli* from the top (sandwich configuration) by carrying out EIS as a function of time during the flow of a 25 µg mL^−1^ Ab solution into the cell. The Nyquist plots are reported in Figure 5a and the corresponding values for *R_ct_* are shown in Figure 5b (see Table S3 for the data) with an exponential fit of the data that provides a constant time of 13.5 ± 0.3 min. Once again, 30 min can be considered a recommended value that allows the accomplishment of the amplification step.

### 3.3. Electrochemical Characterization of the Immunosensor Preparation

Cyclic voltammetry (CV) and electrochemical impedance spectroscopy (EIS) measurements were carried out to investigate the layer by layer construction of the immunosensor and to verify the *E. coli* binding (Figure 6). Both characterization techniques investigate the electron transfer process and, specifically, the changes of charge transfer resistance caused by the adsorption of isolating molecules to the gold electrode. EIS plots of the step-by-step immunosensor fabrication are shown in Figure 6a, utilizing 10 mM Fe(CN)_6_^3−^/Fe(CN)_6_^4−^ as redox probe in 0.01 M PBS buffer.

The potential applied for the EIS studies was set to 0.16 V (vs. Ag/AgCl) according to the CV result. The impedance data are represented as Nyquist plots and *R_ct_* values were extracted by fitting the data with the Randles circuit (inset Figure 6a, see Table S4 for the data),) after each preparation step since the comparison of these values indicate the change of the redox probe kinetics at the electrode interface. The *R_ct_* of the bare electrode was as small as 878 Ω. The surface is functionalized using previously activated antibodies which tether the surface providing a significantly resistance increase of approximately 5.4 kΩ, since the covalent immobilization of antibodies onto the electrode surface acts as an inert electron transfer blocking layer and the penetration of the redox probe. The blocking of the surface was carried out with BSA at 50 µg mL^−1^ as well as at 100 µg mL^−1^ and in both cases a negligible increment of *R_ct_* value could be detected thereby proving that the gold surface is fully covered by the antibodies (Figure S1, see Tables S5 and S6 for the data). In the next step, the solution containing the *E. coli* cells (10^2^ CFU mL^−1^) flows in the circuit and the analyte is recognized by the Abs. However, with the further attachment of the *E. coli* cells (10^2^ CFU mL^−1^) to the modified electrode surface no significant increase of impedance is observed and a solution of anti-*E. coli* Abs is conveyed to the cell giving rise to a sandwich complex which produces an increase of the charge transfer resistance caused by the realization of a further barrier towards the access of the redox probe to the electrode. In such a case, the *R_ct_* value increases by a 15% after the formation of antibody *E. coli* complex.

In addition, CV measurements were carried out to corroborate the EIS results. CV curves of the step-by-step modification are shown in Figure 6b, using 10 mM Fe(CN)_6_^3−^/Fe(CN)_6_^4−^ as redox probe in 0.01 M PBS buffer. The bare gold electrode gave a well-defined anodic and cathodic peaks, due to the reversible interconversion of the redox probe Fe(CN)_6_^3−^/Fe(CN)_6_^4−^, with peak potential difference (Δ*Ep*) of 107 mV and peak current (*Ip*) of 23.9 μA. After antibodies immobilization, the Δ*Ep* increased to 192 mV and the *Ip* decreased to 19.1 μA confirming the attachment of charge transfer inhibiting molecules to the gold electrode. The surface blocking with BSA caused a negligible decrease of *Ip* (18.9) confirming the complete saturation of the gold surface with antibodies. Finally, after the incubation with *E. coli* no significant decrease of current is observed and the resulting formation of antibody-*E. coli* complex lead to a further decrease of the peak current (*Ip* = 17.2 and Δ*Ep* = 218) which coincides with EIS results.

### 3.4. Immunosensor Analytical Performance

**Figure 7:**
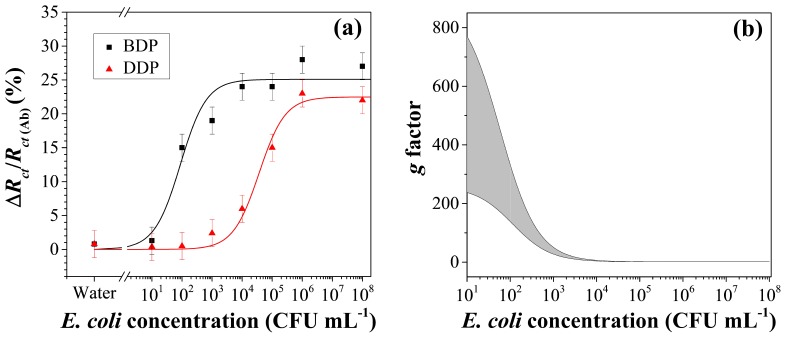
(**a**) Dose-response curve obtained using both protocols: DDP–direct detection protocol and BDP–ballasting detection protocol. The experimental data are fitted by Equation (6); (**b**) Gain factor *g* as a function of the *E. coli* concentration.

The performance of the immunosensor for the detection of *E. coli* was investigated in drinking water by EIS. Drinking water samples (1 mL) were incubated with *E. coli* at different concentration in the range of 10^1^–10^8^ CFU mL^−1^. Figure 7a shows the dose response curves obtained with steps I-III (red curve, direct detection protocol without amplification or DDP) and I-IV (black curve, ballasting detection protocol with amplification or BDP). The sensing parameter is Δ*R_ct_*/*R_ct_*_(Ab)_ where Δ*R_ct_* is the change in the impedance brought about by *E. coli* [in case of DDP Δ*R_ct_* = *R_ct_*_(*E. coli*)_ − *R_ct_*_(BSA)_], whereas *R_ct_*_(Ab)_ is the impedance value measured after the functionalization. As Figure 7a (red curve) shows, a detection range of two decades (10^3^ to 10^5^ CFU mL^−1^) is achieved before reaching a saturation at concentration higher than 10^5^ CFU mL^−1^.

Each concentration has been tested three times in several days in different environmental conditions and using different electrode. The standard deviation (σ) of the measurements is approximately 2% proving that the protocol shows good accuracy and reproducibility. According to the 3σ formula, it is necessary to achieve an impedance increase ≥6% in order to consider the variation as significant. As it shown in the dose-response curve, the limit *E. coli* concentration (LOD) producing such variation in impedance increase is 10^4^ CFU mL^−1^ (Figure 7a, red curve). The Δ*R_ct_*/*R_ct_*_(Ab)_ measured for the negative sample (1 mL aliquot of drinking water) was less than 1% confirming the absence of interferences of the drinking water components (especially salts) with the biosensor surface.

In the DDP condition a LOD of approximately 10^4^ CFU mL^−1^ is obtained, which can be significantly improved by including the step IV that consists of addition of anti-*E. coli* Abs so that a sandwich configuration is realized as shown in Scheme 1 (step IV with Ab II). In fact, the relative electron-transfer resistance difference in such a case is Δ*R_ct_/R_ct_*_(Ab)_ where Δ*R_ct_* = *R_ct_*_(AbII)_ − *R_ct_*_(BSA)_ (Figure 7a, black curve). The black curve shows a remarkable increase in the slope as well as in the saturation level, which is achieved at lower concentration. With the same criteria used before (3σ formula with σ about 2%), the detection limit of this extended protocol (BDP) is estimated to be 3 × 10^1^ CFU mL^−1^ whereas the quantification range is 10^2^–10^3^ CFU mL^−1^. It is worth noticing that a narrow quantification range is not a drawback when “on-off” biosensors are considered, as it is the case of the device proposed here.

The comparison of the two curves in Figure 7a suggests an enhancement of the signal whose factor depends on the *E. coli* concentration. In fact, by defining *g* as a gain factor, we have:

(1) $$g\left(\left[E-coli\right]\right)=\;\frac{\Delta R_{ct}^{\left(BDP\right)}\left(\left[E-coli\right]\right)}{\Delta R_{ct}^{\left(DDP\right)}\left(\left[E-coli\right]\right)},$$

where the superscripts DDP and BDP refer to the three step and four step protocol, respectively. The plot of *g* is reported in Figure 7b as a function of *E. coli* concentration together with the 95% confidence interval (grey area) obtained by propagating the error from the curves in Figure 7a into Equation (1). The enhancement factor is more than one order of magnitude (10 < *g* < 33) at lower concentration and decreases to an expected saturation value of approximately 1 at higher concentrations. Such a behavior may well be explained by considering that at higher concentration of *E. coli*, the surface has a higher degree of occupancy by the bacteria so that the binding of additional Ab II tethered to *E. coli*, gives rise to a smaller effect on *R_ct_* when compared to the effect produced at lower concentration.

The analytical performances of the developed immunosensor have been compared with other recent impedimetric immunosensors for the detection of *E. coli* (Table 1). It is remarkable that all of them share a quite long functionalization time ranging from few hours to even twenty hours to immobilize antibodies on gold electrode surface.

The immunosensors reported in Table 1 are based on immobilization procedures that include the formation of self-assembled monolayers, which usually require particularly smooth gold electrode surfaces [53] and expert personnel for the setup of complex chemical procedures. In contrast, PIT is user-friendly nor is any previous modification of the surface required. Any single electrode (AuSPE) was used “as is” that is without any pretreatment (only a rapid cleaning in H_2_SO_4_) or surface modification, but, even more important, the inherent differences among them in terms of bare impedance did not prevent us from building the more than satisfactory dose-response curve shown in Figure 7a.

### 3.5. Data Fitting

To account for the dose-response curve, we propose a simple model that describes the change of the resistance *R_ct_* due to the analyte recognition as a change of the “effective” electrode area. To start with, let’s consider that while *R_ct_* in the bare electrode is in the range 500–900 Ω, its value increases up to tens of kΩ when the antibodies tether to the surface. Moreover, thanks to our functionalization procedure, no significant change of *R_ct_* is observed after the blocking step (the surface is fully covered by antibodies), and, hence, the initial charge transfer resistance of the biosensor is due to the antibody layer and can be written:

(2) $$R_{ct,0}=\frac{\rho d}{A_{0}}$$

In Equation (2) *ρ* is the resistivity of the antibody layer, *d* its thickness and *A*_0_ the electrode area. Since the contact layer does not change during the detection procedure, both *ρ* and *d* are constant. On the contrary, the occurrence of the analyte detection reduces to some extent the effective area of the electrode, which in turn leads to an increase of the impedance, i.e.:

(3) $$R_{ct}\left(\left[C\right]\right)=\frac{\rho d}{A_{0}-\alpha A\left(\left[C\right]\right)}$$

where A([C]) represents the area occupied by the analytes and *α* is a coefficient that accounts for their conducting properties (α=0, for a “conductive” analyte that does not affect the electrolyte current, whereas α=1 for a fully “insulator” analyte). The occupancy area can be described by the Langmuir isotherm:

(4) $$A\left(\left[C\right]\right)=A_{0}\frac{\left[C\right]}{K+\left[C\right]}$$

where for convenience we used *K* as the inverse of the equilibrium constant. By combining Equations (3) and (4), we have:

(5) $$R_{ct}\left(\left[C\right]\right)=R_{ct,0}\frac{K+\left[C\right]}{K+\left(1-\alpha \right)\left[C\right]}$$

and the sensing parameter becomes:

(6) $$\frac{\Delta R_{ct}}{R_{ct\left(\mathrm{Ab}\right)}}=\frac{R_{ct}\left(\left[C\right]\right)-R_{ct,0}}{R_{ct,0}}=\frac{\left[C\right]}{\frac{K}{\alpha }+\frac{\left(1-\alpha \right)}{\alpha }\left[C\right]}$$

The best fit of the experimental data by the Langmuir-type Equation (6) is shown in Figure 7a, whereas the fitting parameters are reported in Table 2.

As expected, the conductivity coefficient *α*, which measures the microscopic tendency of the analyte to inhibit the electrolyte current, is larger when antibodies are tied to the bacterium. The choice of $\Delta R_{ct}/R_{ct\left(\mathrm{Ab}\right)}$ as sensing parameter not only allows one to measure the values for *α* reported in Table 2, but even more important from the practical point of view, introduces a high degree of robustness in the biosensors as demonstrated by the fact that each experimental point reported in Figure 7a was obtained with different electrode.

The increase of *α* observed when antibodies are bound to bacteria from the top is due to the high “opacity” of the antibodies, the latter property being deducible from the large increment of *R_ct_* brought about by the surface functionalization (see Figure 3, Figure 4, Figure 5 and Figure 6). By imaging that a bacterium fully covered by antibodies would have *α* = 1, we can (under)estimate the fraction *f* of the bacterium area covered by the antibodies as:

(7) $$f=\frac{\alpha_{2}-\alpha_{1}}{1-\alpha_{1}}\approx 0.024$$

In Equation (7) $\alpha_{2}$ and $\alpha_{1}$ correspond to the value of *α* with and without amplification step, respectively. By considering that the area of *E. coli* is $A_{E-c}\approx 1$ µm^2^ and that of an Ab is $A_{Ab}\approx 100$ nm^2^, for the number $N_{Ab}$ of Abs per bacterium, we have:

(8) $$N_{Ab}=f\frac{A_{E-c}}{A_{Ab}}\approx 240$$

Since this calculation is based on the assumption that an antibody is fully “opaque” to the electrolyte current, such a value has to be meant as an underestimation for the number of antibodies binding the bacterium from the top, which is likely to be larger than one thousand.

### 3.6. Immunosensor Specificity

**Figure 8:**
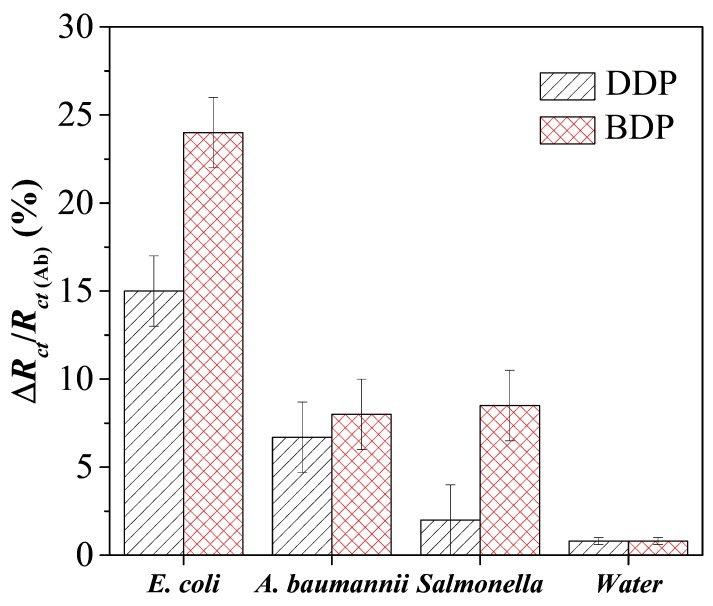
Specificity of the immunosensor in 10 mM Fe(CN)_6_^3−/4−^ solution at pH 7.4. Δ*R_ct_*/*R_ct_*_(Ab)_ induced by 10^5^ CFU mL^−1^ *E. coli* in comparison with the negative control (drinking water) and non-target bacteria (*Acinetobacter baumannii* and *Salmonella enteriditis* 706 RIVM*).*

In order to evaluate the specificity of the developed immunosensor for *E. coli*, we tested the response of the functionalized immunosensor by measuring Δ*R_ct_*/*R_ct_*_(Ab)_ induced by some non-specific bacteria such as *Acinetobacter baumannii* and *Salmonella enteriditis* 706 RIVM. To this end a 1mL aliquot of drinking water incubated with *Salmonella enteriditis* 706 RIVM and *Acinetobacter baumannii* (10^5^ CFU mL^−1^) flowed into the circuit for 30 min at room temperature, followed by a fresh solution of anti-*E. coli* Abs. Figure 8 shows the electron transfer resistance changes for both protocols. According to the direct detection protocol (DDP), Δ*R_ct_*/*R_ct_*_(Ab)_ increased by 2% and 7% when *Salmonella enteriditis* 706 RIVM and *Acinetobacter baumannii* were assayed, respectively, compared with an 15% increase achieved with *E. coli*. Actually, a greater increase with *Salmonella enteriditis* 706 RIVM would be expected in view of the stronger morphological similarities with *E. coli* but, this unexpected behaviour can be likely ascribed to the different shape of *Acinetobacter baumannii*, which is a short, rod-shaped almost round bacterium which mostly hinder at the same concentration. With reference to the extended protocol (BDP), after the ballasting with a fresh solution of anti-*E. coli* Abs, Δ*R_ct_*/*R_ct_*_(Ab)_ increased less than a 9% for both *Salmonella enteriditis* 706 RIVM and *Acinetobacter baumannii*, compared to the marked increase (24%) obtained with *E. coli* cells. This means that the amplification factor is about 1% for *Acinetobacter baumannii* and 4.5% for *Salmonella enteriditis* 706 RIVM, a difference likely due to the superior affinity between anti-*E. coli* pAbs and *Salmonella*, which share the same morphology. These results are more than satisfactory since only a slight cross-reaction arises with other bacterial species.

## 4. Conclusions

An electrochemical impedance immunosensor based on a screen printed gold electrode for the rapid detection of *E. coli* in drinking water is proposed. Firstly, the antibodies were immobilized on the gold electrode surface in a covalent way using the Photochemical Immobilization Technique and the change in charge transfer resistance was monitored in the redox probe Fe(CN)_6_^3−^/Fe(CN)_6_^4−^ using the EIS. The proposed immunosensor exhibits a limit of detection of 3 × 10^1^ CFU mL^−1^ which was obtained by including in the measurement procedure a simple and rapid ballasting step, the latter consisting in the flowing of a fresh antibody solution onto the electrode surface so to realize a sandwich-complex.

PIT is an alternative method to bind antibodies onto gold surfaces and this is the first time it was used in biosensing by EIS. Our results demonstrate that PIT is effective proved even on commercial cheap electrodes This is major achievement since in most situations careful surface treatments are required in order to get an effective sensor response. In addition, PIT is a really a quick functionalization method since only 30 s are required for the antibodies activation and 1 h for the whole functionalization procedure (solution flowing on the electrode surface). Thus, the whole measurement can be carried out in less than 6 h (which means within a working day) since the total analysis time is 3 h and 30 min including washing and sensing processes making our approach suitable for out-of-lab use when low contamination levels need to be detected for alert emergency and, hence, an “on-off” approach is more desirable than a time-consuming quantitative procedure.

## Figures and Tables

**Table 1:** Overview of the latest impedimetric biosensors for *Escherichia coli* detection.

Functionalization Scheme	Functionalization Time (h)	LOD (CFU mL^−1^)	Reference
Au-MHDA-Ab	18 *	2	[44]
Au-Cys-Ferrocene-Ab	20 *	3	[45]
Au-MUA/UDT-Ab	20 *	100	[46]
Au-PrG thiol-Ab	10	140	[47]
Au-AuNPs-PrG thiol-Ab	24 *	48	[47]
Au-PANI-Glu-Ab	>2	100	[48]
Au-Ab (PIT activated)	1	30	This work

Ab: antibody; MHDA: 16-mercaptohexadecanoic acid; Cys: Cysteamine; AuNPs: gold nanoparticles; PrG: Protein G; PANI: polyaniline; Glu: Glutaraldehyde; MUA: 11-mercaptoundecanoic acid; UDT: 1-undecanethiol. * These times have been evaluated by considering overnight as 16 h.

**Table 2:** Fitting parameters *α* and *K* obtained for both protocols DDP and BDP.

	α	K
DDP	0.185 ± 0.007	(3.0 ± 0.7) × 10^4^
BDP	0.20 ± 0.01	70 ± 26

## Supplementary Materials

The following are available online at https://www.mdpi.com/1424-8220/20/1/274/s1, Figure S1: (a) EIS spectrum measured with 100 µg mL^−1^ BSA solution. (b) EIS spectrum measured at different times while a 50 µg mL^−1^ BSA solution is conveyed to the cell, Table S1: Results from the fitting of impedance data (Figure 3) to the Randles circuit, Table S2: Results from the fitting of impedance data (Figure 4) to the Randles circuit, Table S3: Results from the fitting of impedance data (Figure 5) to the Randles circuit, Table S4: Results from the fitting of impedance data (Figure 6) to the Randles circuit, Table S5: Results from the fitting of impedance data (Figure S1a) to the Randles circuit, Table S6: Results from the fitting of impedance data (Figure S1b) to the Randles circuit.
