# Supplementary material for: Screen Printed Based Impedimetric Immunosensor for Rapid Detection of Escherichia coli in Drinking Water
Source: Sensors (Basel). 2020 Jan 3;20(1):274. doi: 10.3390/s20010274 (PMC6982893; doi:10.3390/s20010274)
Supplement: Supplementary file 1 [file sensors-20-00274-s001.pdf]

sensors

Screen Printed Based Impedimetric Immunosensor for Rapid Detection of *Escherichia coli* in Drinking Water

Martina Cimafonte [1], Andrea Fulgione [2,3], Rosa Gaglione [4], Marina Papaianni [3], Rosanna Capparelli [3], Angela Arciello [4], Sergio Bolletti Censi [5], Giorgia Borriello [2], Raffaele Velotta [1] and Bartolomeo Della Ventura [6,*]

[1] Department of Physics "Ettore Pancini", University of Naples "Federico II", Via Cinthia, 26, 80126 Naples, Italy; martina.cimafonte@unina.it (M.C.); rvelotta@unina.it (R.V.)
[2] Istituto Zooprofilattico Sperimentale del Mezzogiorno, Via Salute, 2, 80055 Portici Naples, Italy; andrea.fulgione@unina.it (A.F.); giorgia.borriello@cert.izsmportici.it (G.B.)
[3] Department of Agriculture, University of Naples "Federico II", Via Università, 133, 80055 Portici Naples, Italy; marina.papaianni@unina.it (M.P.); capparel@unina.it (R.C.)
[4] Department of Chemical Sciences, University of Naples "Federico II", Via Cinthia, 26, 80126 Naples, Italy; rosa.gaglione@unina.it (R.G.); angela.arciello@unina.it (A.A.)
[5] Cosvitech Società Consortile a responsabilità limitata, 80142 Naples, Italy; sergiobolletti@cosvitec.eu
[6] Department of Physics, Politecnico di Milano, Piazza Leonardo da Vinci, 32, 20133 Milano, Italy
* Correspondence: dellaventura@fisica.unina.it

Table S1. Results from the fitting of impedance data (Figure 3) to the Randles circuit.

	Rs (Ω)	CPE		Rct (Ω)	W (KΩ/s$^{1/2}$)
		Q_1 μ(s^{n1}/Ωcm^2)	n_1		
Bare AuSPE	253 ± 4	0.339 ± 0.024	0.898 ± 0.008	712 ± 7	3.528 ± 0.042
Ab 15 min	259 ± 3	0.203 ± 0.006	0.917 ± 0.004	2813 ± 14	4.568 ± 0.105
Ab 30 min	256 ± 2	0.192 ± 0.005	0.915 ± 0.003	3384 ± 13	4.694 ± 0.108
Ab 45 min	259 ± 2	0.170 ± 0.003	0.920 ± 0.002	3736 ± 15	4.606 ± 0.097
Ab 60 min	254 ± 2	0.171 ± 0.003	0.919 ± 0.002	3777 ± 13	4.622 ± 0.092

Table S2. Results from the fitting of impedance data (Figure 4) to the Randles circuit.

	Rs (Ω)	CPE		Rct (Ω)	W (KΩ/s$^{1/2}$)
		Q_1 μ(s^{n1}/Ωcm^2)	n_1		
Bare AuSPE	260 ± 7	0.576 ± 0.085	0.850 ± 0.016	787 ± 18	3.978 ± 0.095
Ab	267 ± 2	0.213 ± 0.005	0.919 ± 0.003	4731 ± 24	6.468 ± 0.162
BSA	268 ± 2	0.206 ± 0.004	0.922 ± 0.002	4817 ± 14	6.218 ± 0.162
E. coli 15 min	267 ± 2	0.217 ± 0.004	0.918 ± 0.001	5138 ± 23	6.390 ± 0.160
E. coli 30 min	269 ± 2	0.219 ± 0.004	0.916 ± 0.002	5442 ± 22	6.406 ± 0.160
E. coli 45 min	268 ± 2	0.216 ± 0.004	0.918 ± 0.002	5457 ± 24	6.630 ± 0.165
E. coli 60 min	267 ± 2	0.216 ± 0.004	0.917 ± 0.002	5348 ± 21	6.201 ± 0.150

Table S3. Results from the fitting of impedance data (Figure 5) to the Randles circuit.

	Rs (Ω)	CPE		Rct (Ω)	W (KΩ/s$^{1/2}$)
		Q_1 μ(s^{n1}/Ωcm^2)	n_1		
Bare AuSPE	259 ± 8	0.576 ± 0.086	0.844 ± 0.017	792 ± 18	3.885 ± 0.093
Ab	266 ± 3	0.224 ± 0.009	0.915 ± 0.004	3131 ± 22	5.489 ± 0.154
BSA	266 ± 3	0.223 ± 0.008	0.915 ± 0.002	3149 ± 16	5.642 ± 0.164
E. coli	267 ± 2	0.197 ± 0.005	0.925 ± 0.003	3565 ± 18	5.174 ± 0.124

Ab (II) 15 min	265 ± 2	0.202 ± 0.005	0.922 ± 0.003	3769 ± 19	5.294 ± 0.127
Ab (II) 30 min	265 ± 2	0.201 ± 0.005	0.922 ± 0.003	3939 ± 20	5.400 ± 0.130
Ab (II) 45 min	265 ± 2	0.204 ±0.005	0.920 ± 0.003	3991 ± 20	5.468 ± 0.131
Ab (II) 60 min	265 ± 2	0.205 ± 0.005	0.919 ± 0.003	4044 ± 20	5.605 ± 0.134

Table S4. Results from the fitting of impedance data (Figure 6) to the Randles circuit.

	Rs (Ω)	CPE		Rct (Ω)	W (KΩ/s$^{1/2}$)
		Q$_1$ µ(s^{n1}/Ωcm^2)	n$_1$		
Bare AuSPE	224 ± 1	0.438 ± 0.010	0.892 ± 0.003	878 ± 3	2.837 ± 0.017
Ab	222 ± 1	0.302 ± 0.004	0.889 ± 0.001	5414 ± 16	3.836 ± 0.099
BSA	221 ± 1	0.304 ± 0.003	0.888 ± 0.001	5472 ± 16	3.835 ± 0.099
E. coli	221 ± 1	0.306 ± 0.003	0.887 ± 0.001	5540 ± 14	3.928 ± 0.098
Ab (II)	221 ± 1	0.291 ± 0.003	0.893 ± 0.001	6096 ± 18	3.916 ± 0.117

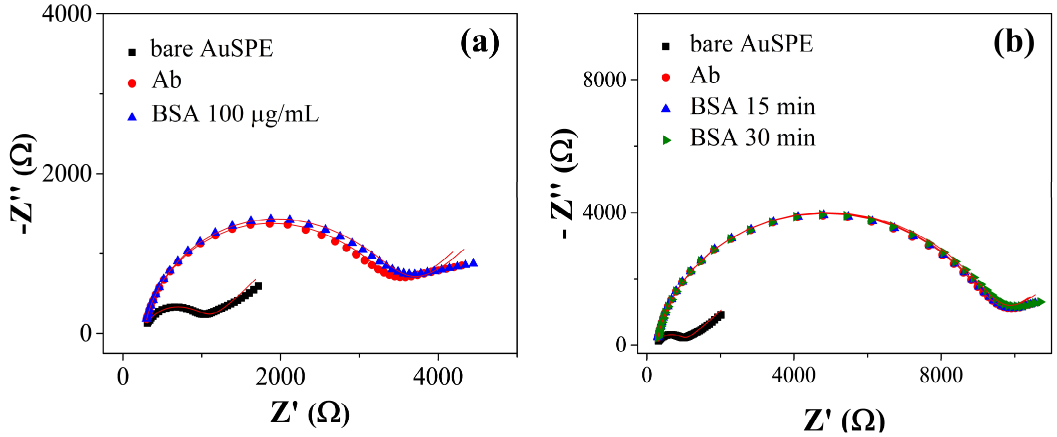

Figure 1. (**a**) EIS spectrum measured with 100 µg mL^{-1} BSA solution. (**b**) EIS spectrum measured at different times while a 50 µg mL^{-1} BSA solution is conveyed to the cell.

Table S5. Results from the fitting of impedance data (Figure S1a) to the Randles circuit.

	Rs (Ω)	CPE		Rct (Ω)	W (KΩ/s$^{1/2}$)
		Q$_1$ µ(s^{n1}/Ωcm^2)	n$_1$		
Bare AuSPE	250 ± 8	0.576 ± 0.087	0.844 ± 0.017	792 ± 18	3.885 ± 0.094
Ab	266 ± 3	0.224 ± 0.009	0.915 ± 0.004	3031 ± 21	5.489 ± 0.153
BSA (100 µg mL^{-1})	266 ± 3	0.223 ± 0.009	0.915 ± 0.004	3149 ± 22	5.642 ± 0.164

Table S6. Results from the fitting of impedance data (Figure S1b) to the Randles circuit.

	Rs (Ω)	CPE		Rct (Ω)	W (KΩ/s$^{1/2}$)
		Q$_1$ µ(s^{n1}/Ωcm^2)	n$_1$		
Bare AuSPE	255 ± 6	0.574 ± 0.069	0.851 ± 0.014	736 ± 14	3.735 ± 0.071
Ab	266 ± 2	0.159 ± 0.002	0.921 ± 0.002	8825 ± 35	5.085 ± 0.239
BSA 15 min	265 ± 2	0.158 ± 0.002	0.921 ± 0.002	8855 ± 35	5.120 ± 0.246
BSA 30 min	266 ± 2	0.164 ± 0.003	0.918 ± 0.002	8919 ± 36	5.373 ± 0.252
